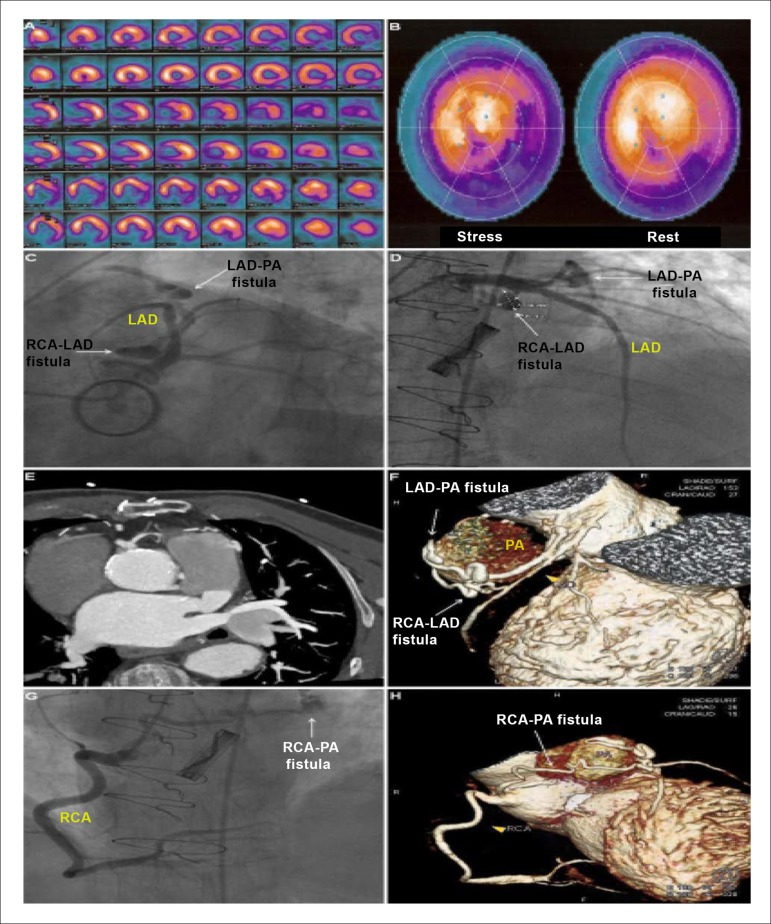# Arteriovenous and Intercoronary Fistulae Presenting as Heart Failure in an Adult

**DOI:** 10.5935/abc.20150139

**Published:** 2015-11

**Authors:** Emmanouil Petrou, Chrysafios Girasis

**Affiliations:** Division of Cardiology, Onassis Cardiac Surgery Center, Athens – Greece

**Keywords:** Heart Failure, Arteriovenous Fistula, Heart Valve Diseases/ surgery

A 79-year-old man with prior aortic valve replacement was admitted to our hospital due to exertional angina. Single-photon emission computed tomography revealed ischemia at the territory of the left anterior descending artery (LAD) and the right coronary artery (RCA) (Figures 1 A and B). Coronary arteriography (Figures 1 C and D) and cardiac computed tomographic angiography (Figure 1 E), including volume rendering reconstruction (Figure 1 F), revealed an arteriovenous fistula between the proximal LAD and the pulmonary artery (PA). There was another fistula between the ostium of the RCA and the PA (Figures 1 G and H). This vessel gave origin to a small branch to the middle part of the LAD, before reaching the PA, thus forming a third, intercoronary fistula (Figures C, D and F).

**Figure:**